# Comparative Study on the Freeze–Thaw Stability of Sodium Caseinate Emulsion-Filled Konjac Glucomannan/κ-Carrageenan Composite Gels

**DOI:** 10.3390/gels11120961

**Published:** 2025-11-28

**Authors:** Weifeng Chen, Guanchen Wu, Lanlan Zhang, Lihua Zhang, Bakht Ramin Shah, Wei Xu

**Affiliations:** 1 School of Food and Bioengineering, Henan University of Animal Husbandry and Economy, Zhengzhou 450046, China; zhuimeng860209@163.com; 2 College of Life Science, Xinyang Normal University, Xinyang 464000, China; w924854122@163.com (G.W.); 15139679341@163.com (L.Z.); lihuazhang25@163.com (L.Z.); 3 DRIFT-FOOD Centre, Faculty of Agrobiology, Food and Natural Resources, Czech University of Life Sciences Prague, 16500 Prague, Czech Republic; raminshah83@gmail.com; 4 Dabie Mountain Laboratory, Xinyang 464000, China

**Keywords:** konjac glucomannan, κ-carrageenan, freeze–thaw stability, emulsions

## Abstract

The paper explored the impact of sodium caseinate (CAS) emulsion on the freeze–thaw stability of konjac glucomannan (KGM)/κ-carrageenan (KC) composite gels. It found that the emulsion and KGM both increased the viscoelasticity of the composite gel, giving it a greater elastic stress. Emulsion addition enhanced the water-holding capacity (WHC) of the KC gel from 72.36% to 89.34%. KGM addition further improved WHC to 97.54%. The hardness of the emulsion KGM/KC gel reached 9.35 N, while the values were essentially not affected by freeze–thaw. This study shows that CAS emulsion, especially under the regulation of KGM and KC, can improve the freeze–thaw stability of the gel system. The results show that emulsion has great potential in regulating the physical and textural properties of multiphase gels. The emulsion coupling method could effectively enhance the freeze–thaw stability of gels, which may provide a new strategy for the development of frozen multiphase gel foods.

## 1. Introduction

Emulsion gel has a three-dimensional network structure filled with oil droplets, and is usually formed by protein, polysaccharides, and other biological macromolecular polymers. It shows great advantage in adjusting food texture for its controllable viscoelasticity. In addition, emulsion gel could be used to load hydrophilic and lipophilic functional substances simultaneously [1,2,3]. For example, emulsion gels stabilized with oleanolic acid exhibited excellent stability when exposed to high temperatures and long-term storage (30 days). When the gels are used as carriers for the co-delivery of curcumin and epigallocatechin gallate. They provided excellent protection for them against light and heat-induced degradation, as well as in the simulated gastric fluids [4]. Additionally, emulsion gels are frequently used as substitutes for animal fat, which can reduce the health risks of excessive intake of saturated and trans fatty acids [5]. Compared to gels, the addition of emulsion can give the emulsion gel a better fat flavor. This make it more applicable as compared to other fat substitutes [6,7,8]. It is known that the emulsion filling had significant impacts on the properties of the gel, including water-holding capacity, rheology, microstructure, etc., while the comparative studies in the processing unit between emulsion gel and gel have been limited.

Currently, freezing treatment is commonly used to extend the shelf life of food or drugs in the food processing and pharmaceutical industries. However, during transportation and storage, the environmental temperature fluctuates repeatedly around the freezing point, causing the products to undergo multiple freeze–thaw cycles, which results in gel deterioration and a decline in product quality [9,10]. During the freezing process, the growth of ice crystals disrupts the network structure within the gel, which is the primary factor causing the deterioration of the gel [11,12]. Enhancing the freeze–thaw stability of the gel is an urgent issue that needs to be addressed. Current studies have shown that adding emulsions is a promising approach to reduce the size of ice crystals and regulate the freeze–thaw stability of gels. The quinoa protein Pickering emulsion can enhance the cross-linking degree of the myofibrillar protein gel network structure by reducing the size of ice crystals generated during freezing, thereby improving the gel strength and water retention capacity [13,14]. Pickering emulsion gels stabilized by soy protein cross-linking through transglutaminase enzyme exhibited excellent freeze–thaw stability and could be applied to frozen foods [15,16]. Although the emulsion gel displayed favorable freeze–thaw stability. However, previous studies commonly research the freeze–thaw stability of emulsion or emulsion gel individually.

Konjac glucomannan (KGM), formed by the connection of glucose and mannose through β-1-4 bonds, possesses strong gelation ability, swelling ability, and high viscosity [17,18,19]. In addition, KGM also possesses prominent functional characteristics, such as anti-obesity, anti-tumor, anti-diabetes, and cholesterol reduction [20,21]. Due to its excellent properties, KGM is widely used in the food industry. KGM enables the emulsions to generate higher viscoelasticity and a stronger gel network. It inhibits the damage to Pickering emulsions caused by thermal treatment and enhances the thermal stability of the emulsions [22]. Other studies have shown that KGM increases the retention rate of β-carotene by increasing the thickness of the interface film and improving the bioavailability of β-carotene [23]. KC, extracted from seaweed, is a naturally extracted linear anionic polysaccharide. As a temperature-sensitive polysaccharide, KC can form a thermoreversible hydrogel and display excellent thickening and stability [24]. Studies have shown that KC has the potential to regulate emulsions. It demonstrated that the porous network structure formed by KC in the continuous phase could effectively separate the oil droplets from each other and maintain the stability of the Pickering emulsion [25]. However, the single KC gel has the drawbacks of high brittleness and high hardness, which cannot meet the application requirements. The synergistic effect of KGM and KC may effectively improve the adverse properties of KC, such as high brittleness, easy dehydration, contraction, etc. [26,27,28]. The research showed that the composite hydrogel with a mixing ratio of KGM and KC of 1:9 had a good sustained release effect on glucose [29].

Therefore, based on our previous research about the camellia oil emulsion stabilized by sodium caseinate (CAS). In this paper, our aim is to use the emulsion as a filler in the KGM/KC composite gel. After several freeze–thaw treatments, the effects of emulsion on gel are systematically researched through water-holding capacity, water distribution, textural analysis, rheological analysis, and microstructure observation. The mechanism of freeze–thaw stability regulated by emulsion was preliminarily discussed. The regulation of emulsion on gel may provide a new perspective for exploring frozen foods.

## 2. Results and Discussion

### 2.1. Water-Holding Capacity of Emulsion Gel with Different Freeze–Thaw Cycles

**Figure 1:**
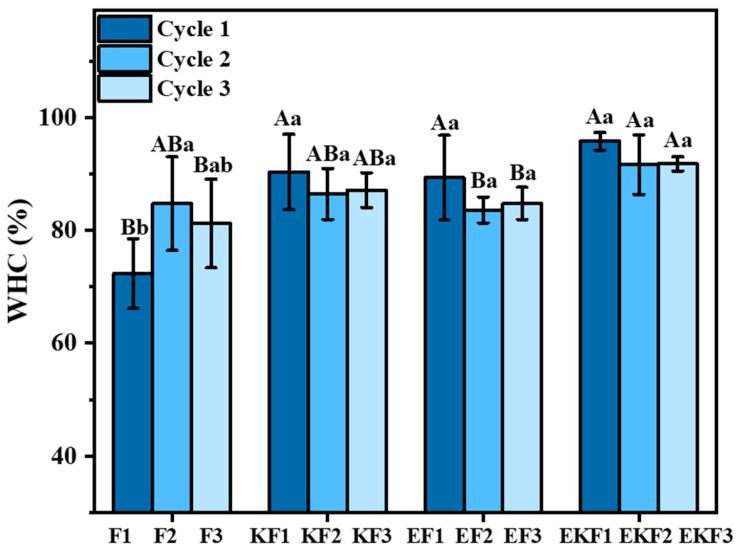
Water-holding capacity of emulsion gel with different freeze–thaw cycles. Different lowercase and capital letters indicate significant differences under component and freeze–thaw treatment (*p* < 0.05).

Water-holding capacity (WHC) is an important parameter for gel quality under different freeze–thaw cycles. It reflects the strength of the interaction between the three-dimensional network and water [30]. It can be seen that the WHC of F1, F2, and F3 was 72.36%, 84.74%, and 81.23%, respectively, after three freeze–thaw cycles (Figure 1). After adding KGM, the WHC of emulsion gel increased to 90.37%, 86.45%, and 87.13%, respectively. This is because the KGM, which is rich in hydrophilic hydroxyl and carboxyl groups, can form hydrogen bonds with water molecules in the aqueous phase, thereby enhancing the WHC of gels. Similar to the previous study, KGM made the three-dimensional network structure of the gel denser, thereby reducing the degree of water separation from the network after freeze–thaw [31]. The WHC of the gel increased after emulsion addition, and no significant reduction was observed after freeze–thaw cycle. This resulted from the oil droplets encapsulated by CAS molecules being uniformly dispersed in the three-dimensional network structure formed by KGM. Another important reason was that the water retention ability of the gel mainly depended on capillary force, which was enhanced in the presence of oil droplets [32]. As freeze–thaw cycles increased, the WHC of F showed a downward trend. This was because the ice crystals damaged the three-dimensional network structure within the gel and weakened the water-capturing ability of the emulsion gel.

### 2.2. Rheological Behaviors of Emulsion Gel with Different Freeze–Thaw Cycles

**Figure 2:**
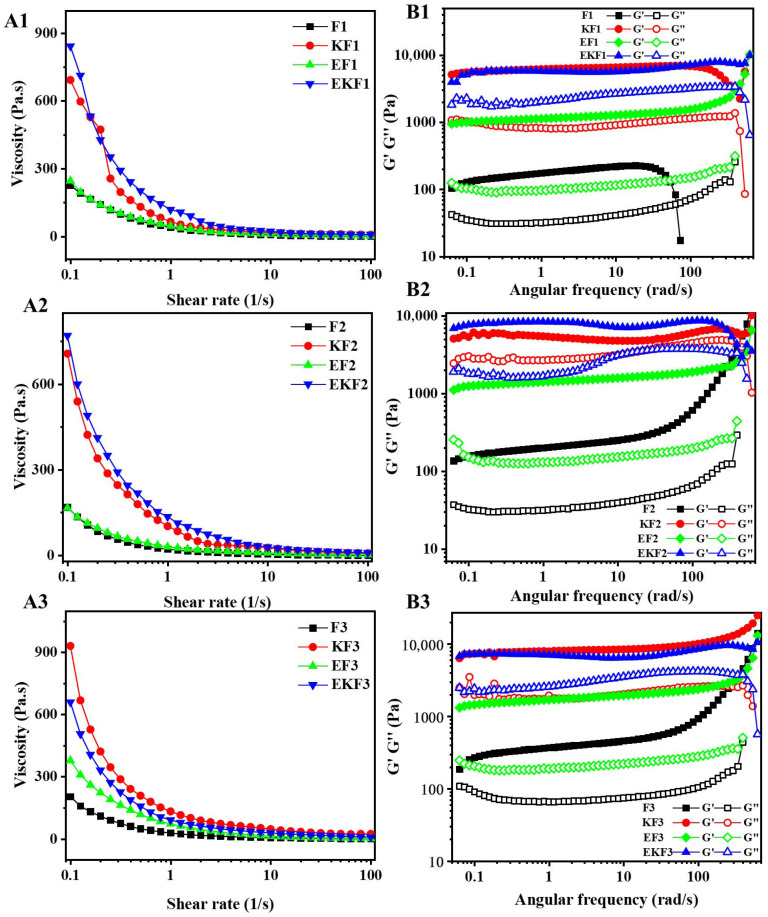
Flow sweeps (**A**) and frequency sweeps (**B**) of emulsion gel with different freeze–thaw cycles, the numbers (1, 2 and 3) in the figures indicate freeze–thaw cycles.

Figure 2 displayed the apparent viscosity of the gel and emulsion gel with different freeze–thaw cycles. All samples exhibited a significant shear thinning behavior within the shear rate range of 0.1–100 s^−1^. It noted that KGM led to a significant increase in the initial apparent viscosity of the emulsion gel. The frequency sweep curves showed that the G′ of emulsion gels is greater than G′′. The emulsion gels showed obvious gel characteristics in freeze–thaw the process. The dependence of G′ on frequency reflects the strength of the gel. The G′ and G′′ of all emulsion gels were almost parallel within the frequency range and had a slight frequency dependence, reflecting that the three-dimensional network of the gel has good viscoelasticity. This results were consistent with the results of WHC.

**Figure 3:**
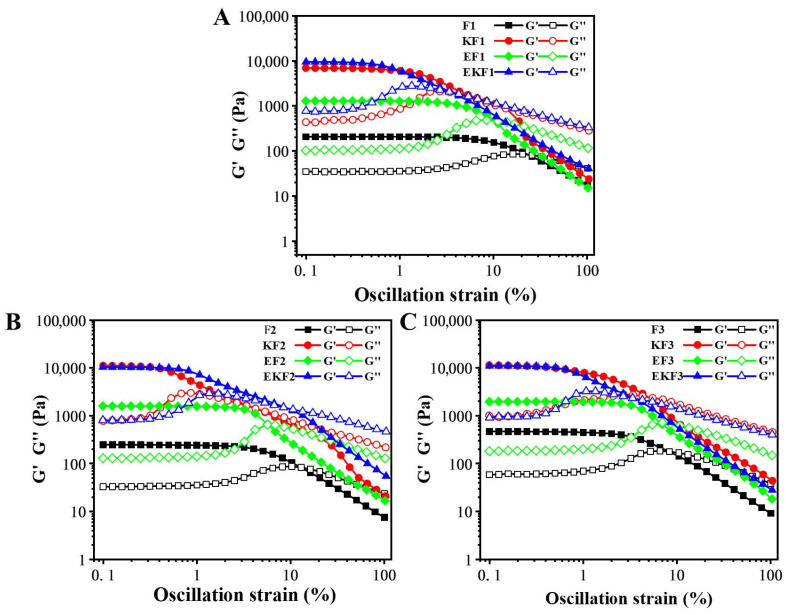
Oscillation amplitude sweeps of emulsion gel with different freeze–thaw cycles (**A**–**C**) indicated the freeze–thaw cycles were 1, 2, and 3.

Extensive oscillation sweep (LAOS) reflects the molecular interactions and viscoelastic behavior. It was used to study the structural change in emulsion gels during freeze–thaw cycles. As shown in Figure 3, G′ and G′′ initially exhibited a parallel linear state. This interval was called the linear viscoelastic region (LVR), and the intersection point of G′ and G′′ was the critical strain (γc). The larger γc and the longer LVR indicated that the material had a stronger ability to maintain its initial structure under large deformations [33]. After γc, both G′ and G′′ showed significant decreases. Before the decrease, G′′ exhibited a small increasing trend that was referred to as type III nonlinear behavior [34]. After the KGM addition, the rigidity of the gel becomes stronger due to the strong hydrogen bond interactions. The γc value of KF1 is 1.02%, and the value was 1.07% for EKF1. While the γc value decreased to 0.85% and 0.51% for KF3 and EKF3. With the increase in freeze–thaw cycles, the γc of all samples decreased, and the LVR became shorter, indicating the structure of the emulsion gel was disrupted. For EKF, G′ significantly increased after freeze–thaw cycles. It notes that the G′ value is always higher than that of KF, K, and EK. This also illustrated that SA emulsion filling improved the solid properties of KGM/KC gel under different freeze–thaw cycles. The increase in G′′ was attributed to the fact that the microstructure of the three-dimensional network was damaged during the oscillation process. Therefore, the structure was prone to being damaged under larger forces, resulting in smaller γc and shorter LVR.

**Figure 4:**
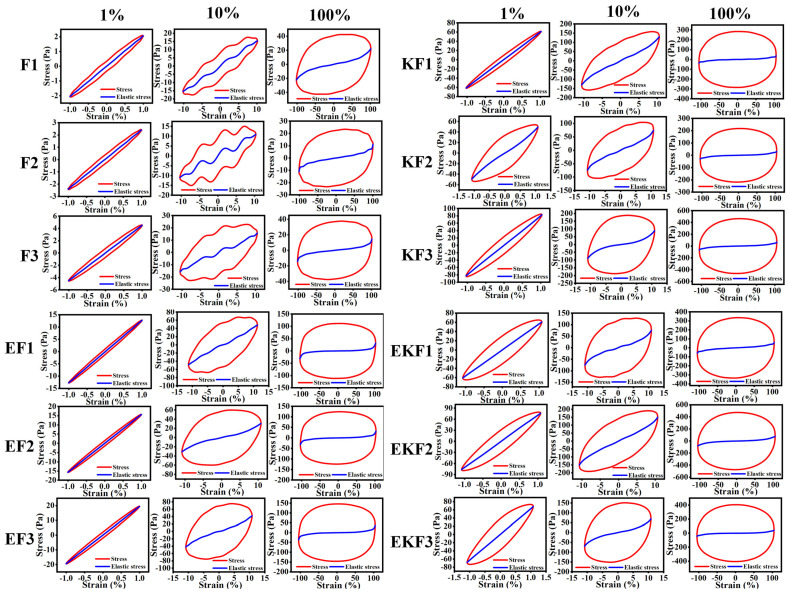
Lissajous plots of emulsion gels with different freeze–thaw cycles and different compositions at different strains (1%, 10%, 100%).

In order to conduct a more in-depth study on the nonlinear viscoelastic behavior, Lissajous diagrams were further analyzed LAOS of emulsion gels with freeze–thaw cycles (Figure 4). The stress response in the elastic Lissajous curve of LVR was a narrow ellipse, and an increase in the area of the ellipse indicated material softening. At the low strain (1%), the stress of all samples exhibited a narrow elliptical shape, indicating an elastic-dominated behavior [35]. As the strain increased, the curve area expanded, and the shape gradually changed from a narrow elliptical shape to a rounded rectangle, suggesting the internal behavior of the emulsion gel shifted from elastic to viscous. KF and EKF exhibited good viscoelastic properties with significant elastic stress peaks. After three freeze–thaw cycles, the elliptical areas all increased. It indicated the freeze–thaw cycles had caused damage to the structure of the gels [36]. In contrast, F showed significant changes in the Lissajous curve after freeze–thaw cycle. The elastic stress was distorted, which indicated that damage occurred to the internal structure under large strains. These results suggested that KC gel was damaged after multiple freeze–thaw cycles.

### 2.3. TPA Analysis of Emulsion Gel with Different Freeze–Thaw Cycles

**Figure 5:**
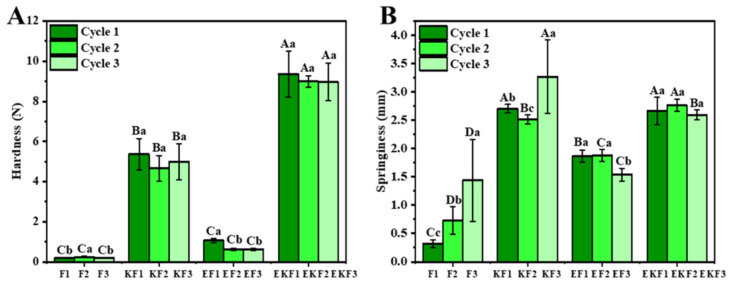
Hardness (**A**) and springiness (**B**) of emulsion gels with different freeze–thaw cycles. Different lowercase and capital letters indicate significant differences under component and freeze–thaw treatment (*p* < 0.05).

The texture properties of emulsion gels with different freeze–thaw cycles were evaluated through hardness and elasticity (Figure 5). Except for F, the hardness of all samples deteriorated after freeze–thawing. During the freeze–thaw cycles, the growth of ice crystals enlarged the pores inside the gel and disrupted the continuity of the network structure. The gel lost water due to freeze–thawing, resulting in a decrease in the overall water content of the system. The gel network structure became dense again and caused the hardness of the gel to recover after the third freeze–thaw treatment. Moreover, the freeze–thaw cycle disrupted the gel structure, which resulted in a honeycomb-like hollow structure inside [37]. This also explained the increase in the elasticity of F in Figure 5B. Previous studies had shown that KGM and KC can interact to form a dense and stable three-dimensional network structure. So KF and EKF had excellent texture properties. The hardness and elasticity of EF gel decreased after multiple freeze–thaw cycles, which was caused by the aggregation of protein groups. The same phenomenon also occurred in EKF gel, but there was no significant change in the hardness and elasticity of EKF gel.

### 2.4. Water Distribution of Emulsion Gel with Different Freeze–Thaw Cycles

**Figure 6:**
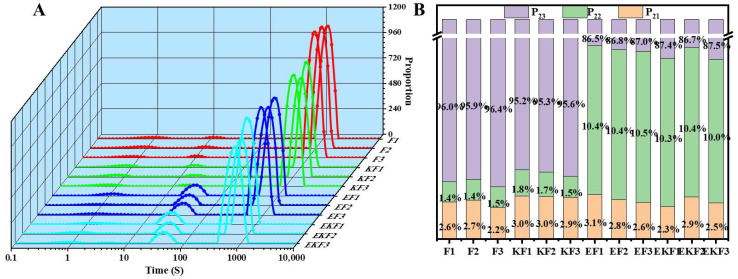
T_2_ relaxation curves (**A**) and content (**B**) of emulsion gel with different freeze–thaw cycles.

Low-field nuclear magnetic resonance was used to determine the water distribution in emulsion gel. The T2 relaxation time of the gels exhibited three peaks (Figure 6), representing water bound to the macromolecule (0–10 ms, T_21_), immobilized water trapped within the gel network (40–180 ms, T_22_), and free water (500–2000 ms, T_23_). With the increase in freeze–thaw cycles, the content of free water (P_23_) increased. The formation of large ice crystals reduced the stability of water, causing water to migrate to free water. In addition, freeze–thaw cycles damaged the proteins and polysaccharides in the gel and deteriorated its texture. The destruction of the structure led the gel to lose water. After the introduction of emulsion and KGM, the P_21_ and P_22_ of the emulsion gel increased, while P_23_ decreased. This indicates that both emulsion and KGM enhanced the water capture ability of the emulsion gel, inhibited the migration of water after freeze–thaw, and improved the stability of the system [38].

### 2.5. CLSM Analysis of Emulsion Gel with Different Freeze–Thaw Cycles

**Figure 7:**
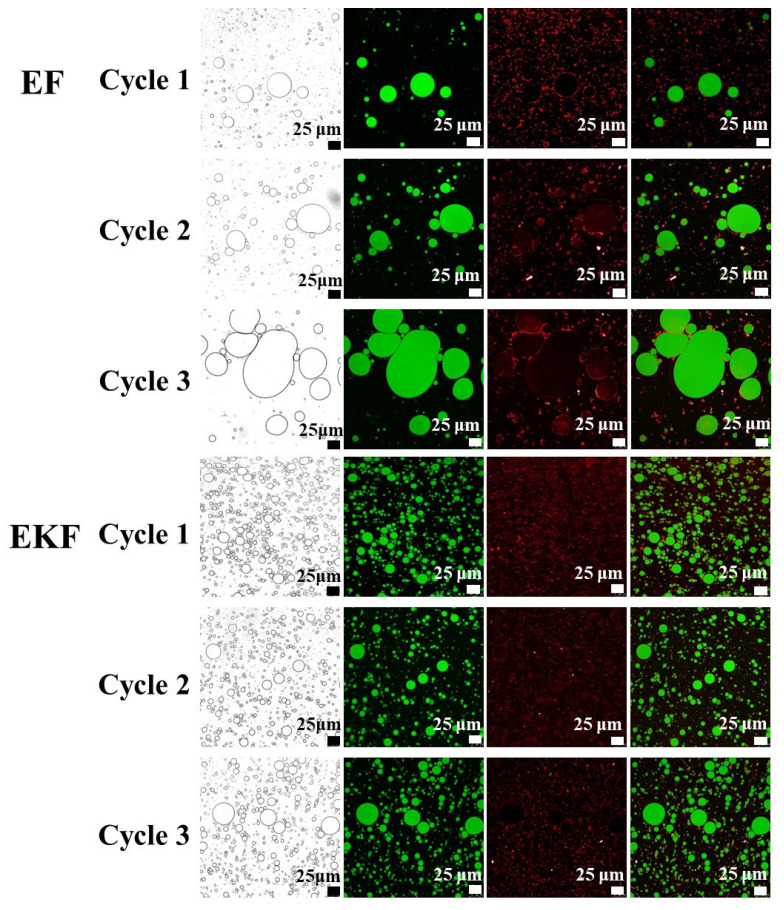
CLSM images of emulsion gels with different times of freeze–thaw cycles. The red and green areas represent the aqueous phase and the oil phase respectively.

To deeply investigate the influence of freeze–thaw cycle on the droplet distribution in the emulsion gel, CLSM was used to observe the microstructure of EF and EKF. Neither F nor KF contains the oil phase, so the internal droplet distribution could not be observed. As shown in Figure 7, the oil phase and CAS in the emulsion gel were stained with Nile Red and Nile Blue, respectively. With the increase in the number of freeze–thaw cycles, the emulsion in the gel broke and aggregated, and the gel structure was damaged. It noted that the oil droplets (green) in both EF and EKF showed a tendency to aggregate. Meanwhile, the CAS (red) wrapped around the oil droplets also showed a certain degree of flocculation. This might be attributed to the fact that the interface membrane formed by the protein breaks down during the freeze–thaw cycle, which disrupts the balance between the aqueous phase and the oil phase, causing the oil droplets to aggregate. It was observed that EF exhibited irregular agglomeration phenomena as the number of freeze–thaw cycles increased. The oil droplets in the EKF were more uniform in size. It was consistent with the previous findings. KGM reduced the damage to the CAS emulsion and protected the emulsion structure.

### 2.6. SEM Analysis of Emulsion Gel with Different Freeze–Thaw Cycles

**Figure 8:**
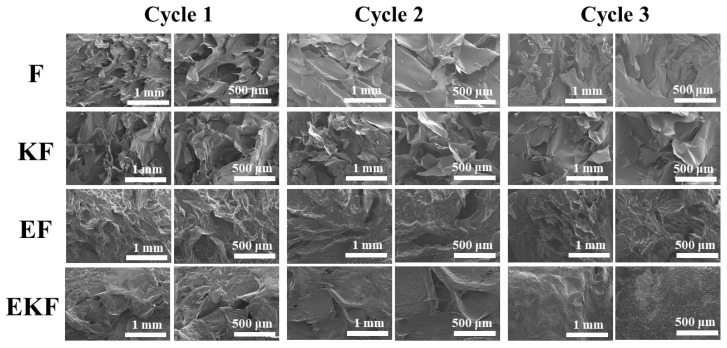
SEM images of emulsion gels with different times of freeze–thaw cycles.

Figure 8 shows the cross-sectional microstructure of the emulsion gel. F presented a loose network structure with large pores. Comparatively, KF had smaller pores, which were consistent with the phenomenon observed in our previous study [38]. The surfaces of EF and EKF were smooth with smaller pores. With the increase in freeze–thaw cycles, the structures of each group of emulsion gel had been damaged to different degrees. The water in the emulsion gel breaks hydrogen bonds at low temperatures and forms ice crystals. During the freeze–thaw cycle, the growth and recrystallization of ice crystals caused the network structure to become loose or even break [39]. Among them, F was the most severely damaged. After the third freeze–thaw cycle, the structure collapsed, and it is difficult to observe a continuous three-dimensional network. EF still had a continuous three-dimensional network structure after the freeze–thaw cycle. The smaller oil droplets filled in the gel matrix to form larger micelles and a denser network structure, which improved the freeze–thaw stability of the emulsion gel. This phenomenon can be further confirmed through the quantitative pore size determined from microscopic images using image analysis software (Figure S1). Appropriate emulsions can delay the formation of ice crystals and maintain the original structure of the gel, reducing the damage to the gel structure caused by freeze–thaw cycles [40]. The dense network structure formed by the interaction of KGM and KC had excellent freeze–thaw stability. In particular, EKF still had a dense structure after multiple freeze–thaw cycles as compared with other samples.

## 3. Conclusions

This paper comparatively investigated the freeze–thaw stability of KGM/KC composite gel after CAS emulsion filling. It indicated that both emulsion and KGM could enhance the gel’s ability to capture water. Emulsion and KGM could maintain the gel’s viscoelasticity and enhance its deformation resistance. KGM addition enabled the gel to maintain strong rigidity after multiple freeze–thaw cycles. Emulsion maintained the gel’s hardness and elasticity after freeze–thaw treatment. These results indicated that emulsion and KGM had a synergistic effect in improving freeze–thaw stability of the composite gel. The emulsion filling strategy shows potential for the development of frozen emulsion-based foods, such as smoothies or cheesecake. The synergistic effect on the freeze–thaw stability of practical frozen emulsion food systems needs further study.

## 4. Materials and Methods

### 4.1. Materials

KGM (Mw 1.4 × 10^6^ Da) is kindly provided by Yizhi Konjac Industry Co. Ltd. (Yichang, China). κ-carrageenan (KC) was purchased from Shanghai Aladdin Biochemical Technology Co., Ltd. (Shanghai, China). Camellia oil was purchased in a local supermarket that was produced by Henan Lvda Mountain Camellia Oil Co., Ltd. (Xinyang, China). CAS, Nile Red, and other chemicals were reagent-grade, purchased from Sinopharm Chemical Reagent Co., Ltd. (Shanghai, China). All solutions in the study were prepared by ultrapure water through a Milli-Q water purification system (Millipore, Burlington, MA, USA).

### 4.2. Preparation and Freeze–Thaw Treatment of Emulsion-Filled Composite Gel

KGM (0.6 wt%) was dissolved in water by stirring for 6 h at 20 °C. Similarly, CAS solution (1 wt%) was prepared under mechanical agitation for 2 h at 25 °C. CAS emulsion was prepared by homogenizing (12,000 rpm, 3 min, 500 W) with camellia oil (20 wt%) as the oil phase using a homogenizer (Ultra Turrax T18, IKA, Staufen, Germany). KC (1 wt%) was slowly dispersed in the above solutions and stirred in a water bath at 70 °C for 20 min. After KC is fully dissolved, all the samples are cooled to room temperature (25 °C) to form a gel. Fresh samples were refrigerated at −18 °C for 22 h, and then thawed in a 30 °C water bath for 2 h. The process is defined as one freeze–thaw cycle. As shown in Table 1, the samples were divided into four groups: F, KF, EF, and EKF. The number of freeze–thaw cycles for each sample was marked with a number at the end of the sample name (such as F1, F2, and F3). Each cycle was conducted independently.

### 4.3. Measurement of Water-Holding Capacity

The water-holding capacity (WHC) was determined using a previous method [41]. The gel (5 g) was placed in a 50 mL plastic centrifuge tube and subjected to high-speed centrifugation at 10,000 rpm for 20 min at 20 °C. And then the supernatant was carefully removed, and the WHC was calculated using the following formula (1):

(1) $$\mathrm{WHC}\;(\%)\;=\;\frac{m_{3}\;-{\;m}_{1}}{m_{2}}\;\times \;100$$

where m_1_ represents the mass of the plastic centrifuge tube (g), m_2_ represents the total mass of gel, and m_3_ represents the total mass of the sample after removing the supernatant (g).

### 4.4. Measurement of Rheological Behavior

The apparent viscosity of emulsion gel was measured through a rotational rheometer (DHR-2, TA, New Castle, DE, USA) using a 20 mm parallel plate with a shear rate of 0.1–100 s^−1^ and 1% strain. A frequency sweep was conducted in the range of 0.1 to 100 Hz with a strain of 0.1%. LAOS measurements were conducted from 0.01% to 100% with a frequency of 1 Hz at 25 °C. Additionally, the raw data for different strains (1%, 10%, and 100%) were collected and then analyzed using the Lissajous diagram of the MITlaos program. Each sample was conducted five times.

### 4.5. Textural Properties Measurement

After equilibrating at room temperature for 30 min, composite hydrogels with or without CAS emulsion were subjected to textural testing using a texture analyzer (TMS-Pro 3000, FTC, Sterling, VA, USA) at 25 °C. In the TPA test, a cylindrical probe (d = 40 mm) was used. The test speed was 20 mm/min, the minimum trigger force was 0.1 N, and the deformation amount was 25%. Each sample was tested five times.

### 4.6. Low-Field Nuclear Magnetic Resonance Measurement

An LF-NMR spectrometer (NMI20-015V-1, Niumag, Suzhou, China) was used to measure the water distribution in gel and emulsion gels. The thawed samples were placed in the nuclear magnetic resonance tubes and the transverse relaxation time was determined in Carr–Purcell–Meiboom–Gill mode. The interval time (TW) is 2000 ms, the delay parameter (TE) is 1.0 ms, the cumulative sampling times (NS) is 8, and the number of echoes (NECH) is 10,000.

### 4.7. Microstructure Observation

The microstructure of composite hydrogels with different freeze–thaw cycles was observed using a confocal laser microscope (SP8, Leica, Wetzlar, Germany) and a scanning electron microscope (SEM) (Hitachi S4800, Tokyo, Japan). For CLSM, the emulsions were dyed with 40 μL Nile Red/Nile Blue A mixed solution (0.1%, *w*/*v*), and then observed under a microscope with excitation light sources at 488 nm and 633 nm. For SEM, the gel was first freeze-dried, sprayed with platinum, and observed at an acceleration voltage of 5 kV.

### 4.8. Statistical Analysis

All experiments were performed five times and reported as mean ± standard deviation. The results were analyzed using One-way ANOVA and Duncan’s multiple comparison tests to identify the significant differences (*p* < 0.05) using SPSS 25.0 software (SPSS Inc., Chicago, IL, USA).

## Figures and Tables

**Table 1:** Freeze–thaw treatment of KGM/KC composite gel filled with CAS emulsion.

Sample	CAS Emulsion	Composite Gel	Number of Freeze–Thaw Cycles
F1	N	0% KGM 1% KC	1
F2	N	0% KGM 1% KC	2
F3	N	0% KGM 1% KC	3
KF1	N	0.6% KGM 1% KC	1
KF2	N	0.6% KGM 1% KC	2
KF3	N	0.6%KGM 1% KC	3
EF1	Y	0% KGM 1% KC	1
EF2	Y	0% KGM 1% KC	2
EF3	Y	0% KGM 1% KC	3
EKF1	Y	0.6% KGM 1% KC	1
EKF2	Y	0.6% KGM 1% KC	2
EKF3	Y	0.6% KGM 1% KC	3

Note: N and Y indicate without and with CAS emulsion.

## Data Availability

The original contributions presented in this study are included in the article/Supplementary Materials. Further inquiries can be directed to the corresponding author.

## Supplementary Materials

The following supporting information can be downloaded at: https://www.mdpi.com/article/10.3390/gels11120961/s1, Figure S1: Quantitative pore size analysis from SEM images of composite gels with different freeze-thaw cycles.
