# Supplementary material for: Comparative Study on the Freeze–Thaw Stability of Sodium Caseinate Emulsion-Filled Konjac Glucomannan/κ-Carrageenan Composite Gels
Source: Gels. 2025 Nov 28;11(12):961. doi: 10.3390/gels11120961 (PMC12732840; doi:10.3390/gels11120961)
Supplement: Supplementary file 1 [file gels-11-00961-s001.zip › gels-3973970-supplementary.pdf]

Supplementary Materials

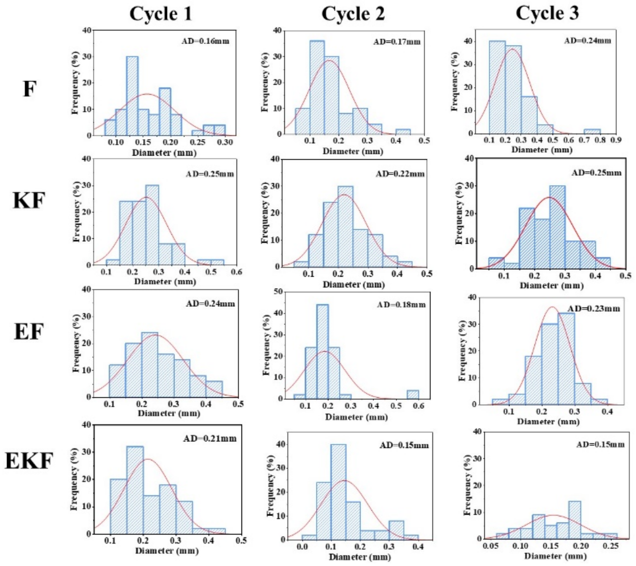

Figure S1 Quantitative pore size analysis from SEM images of composite gels with different freeze-thaw cycles
